# Lysostaphin: A Staphylococcal Bacteriolysin with Potential Clinical Applications

**DOI:** 10.3390/ph3041139

**Published:** 2010-04-19

**Authors:** Maria do Carmo de Freire Bastos, Bruna Gonçalves Coutinho, Marcus Lívio Varella Coelho

**Affiliations:** Departamento de Microbiologia Geral, Instituto de Microbiologia Prof. Paulo de Góes, CCS, Bloco I, UFRJ, 21941-590, Rio de Janeiro, RJ, Brazil, E-mails: coutinhobg@gmail.com (B.G.C.); mcvarella@yahoo.com.br (M.L.V.C.)

**Keywords:** lysostaphin, staphylococci, antimicrobial peptides, bacteriocins, staphylococcins

## Abstract

Lysostaphin is an antimicrobial agent belonging to a major class of antimicrobial peptides and proteins known as the bacteriocins. Bacteriocins are bacterial antimicrobial peptides which generally exhibit bactericidal activity against other bacteria. Bacteriocin production is a self-protection mechanism that helps the microorganisms to survive in their natural habitats. Bacteriocins are currently distributed into three main classes. Staphylococcins are bacteriocins produced by staphylococci, which are Gram-positive bacteria of medical and veterinary importance. Lysostaphin is the only class III staphylococcin described so far. It exhibits a high degree of antistaphylococcal bacteriolytic activity, being inactive against bacteria of all other genera. Infections caused by staphylococci continue to be a problem worldwide not only in healthcare environments but also in the community, requiring effective measures for controlling their spread. Since lysostaphin kills human and animal staphylococcal pathogens, it has potential biotechnological applications in the treatment of staphylococcal infections. *In vitro* and *in vivo* studies performed with lysostaphin have shown that this staphylococcin has potential to be used, solely or in combination with other antibacterial agents, to prevent or treat bacterial staphylococcal infectious diseases.

## 1. Introduction

Bacteriocins are proteinaceous compounds produced by bacteria which generally display bactericidal activity against other bacterial spp. [1]. The bacteriocin-producing strains have developed a protective immunity system against their own bacteriocin. Each bacteriocin has its own immunity system, which is generally expressed concomitantly with the bacteriocin structural genes [1]. 

Bacteriocins produced by Gram-positive bacteria are the most studied ones. They form a heterogeneous group of peptides and proteins. They can be active against other bacteria, belonging to only closely-related species (narrow spectrum) or to different bacterial genera (broad spectrum). Their genetic determinants are generally arranged in the form of operons, which may be encoded on the bacterial chromosome, although they are usually found on plasmids [1]. 

Recently, Bierbaum and Sahl [2] and Nissen-Meyer and co-workers [3] proposed modifications in the classification of the bacteriocins produced by Gram-positive bacteria to accommodate data related to the annual description of a repertoire of bacteriocins, some of them with distinctive characteristics. Based on the data so far available, a classification scheme is presented in Table 1, which is a combination of both classifications, with minor modifications. According to it, bacteriocins produced by Gram-positive bacteria can be grouped into three main classes, all of them with subdivisions. Bacteriocins from classes I and II, which comprise small peptides, occur more frequently and possess potential industrial applications [4,5]. Knowledge of all aspects of bacteriocin biology, including the elucidation of their structure-function relationships, production, immunity, regulation, and mode of action, is required when considering bacteriocin applications. However, for a more comprehensive review on both class I and class II bacteriocins, the reader is referred to recent publications written by Bierbaum and Sahl [2] and Nissen-Meyer and co-workers [5]. 

Among the bacteriocins produced by staphylococci [6], lysostaphin, which is the prototype class III bacteriocin, is the most studied one with regard to clinical applications. Therefore, in the present review, we attempt to provide the reader with an overview of lysostaphin: its structure, relevant features, mode of action, and potential practical applications. 

## 2. Relevant Features of Staphylococci

The genus *Staphylococcus* is composed of Gram-positive cocci arranged in grape-like clusters, divided currently into 42 recognized species and 24 subspecies [7]. They are widespread in nature, being a commensal of the skin, skin glands, and mucous membranes of mammals and birds. Staphylococci generally have a benign or symbiotic relationship with their host. However, they may develop the life-style of a pathogen if they gain entry into the host tissue. Certain *Staphylococcus* species are found frequently as etiologic agents of a variety of human and animal infections [8].

**Table 1:** Classification scheme for bacteriocins produced by Gram-positive bacteria proposed by Sahl & Bierbaum [2] and Nissen-Meyer *et al.* [3], with modifications.

Classification	Relevant Features	Groups/Subclasses	Examples
Class I (lantibiotics)	Small, heat-stable peptides (<5 kDa), containing modified amino acids (lanthionine, 3-methyl-lanthionine, dehydrated amino acids, S-aminovinyl-cystein, among others)	Type A (linear)	Nisin, Pep5, Epidermin,
		Type B (globular)	Mersacidin
		Type C (two components)	Lacticin 3147
		Type D (reduced antimicrobial activity)	SapT
Class II	Small, heat-stable peptides (<10 kDa), containing no modified amino acids	IIa (linear; pediocin-like)	Pediocin PA-1
		IIb (linear; two components)	Lactacin F
		IIc (cyclic peptides)	Enterocin AS-48
		IId (linear)*	Aureocin A53
		IIe (linear; more than two components)	Aureocin A70
Class III	Large, heat-labile proteins	Type IIIa (bacteriolysins)	Lysostaphin
		Type IIIb (non-lytic)	Helveticin J

* This subclass includes a number of bacteriocins with distinctive characteristics such as non pediocin-like processed bacteriocins, bacteriocins with no leader peptides or with *sec*-dependent leaders.

Staphylococci are generally divided in two main groups, coagulase-positive staphylococci (CoPS) and coagulase-negative staphylococci (CoNS) based on the production of coagulase, an enzyme-like factor that causes fibrin to coagulate and form a clot, a trait which is generally associated with pathogenicity [8].

During the last century, *S. aureus*, a CoPS, has become the leading overall cause of nosocomial infections worldwide [8]. *S. aureus* causes a wide range of serious infections, ranging from localized or systemic abscesses, septicemia, and endocarditis to septic emboli. This pathogenicity reflects its ability to produce a variety of toxins, to attach to medical devices by production of biofilms, and to an extraordinary ability to develop antimicrobial resistance [9]. After introduction of the methicillin in 1961 to treat infections by penicillin-resistant *S. aureus,* there were reports of isolation of multi-resistant *S. aureus* strains [8,9]. Currently, strains of methicillin-resistant *S. aureus* (MRSA) are becoming increasingly difficult to combat because of emerging resistance to all current antibiotic classes [10]. MRSA has gradually disseminated and MRSA is now a problem in hospitals worldwide and is increasingly recovered from nursing homes and the community [8,9]. CoNS, such as *S. epidermidis*, *S. haemolyticus, S. lugdunensis*, and *S. saprophyticus*, among others, can also be found in association with human infections. CoNS are the most frequently reported pathogens in nosocomial blood-stream infections, being responsible for 30% to 40% of these infections [11]. They are often associated with implanted medical devices [8,9,11]. *S. saprophyticus* is a frequent cause of urinary tract infections in young women [8,11]. Increasing drug-resistance among CoNS has also become of concern [8,11]. Therefore, the rising level of resistance to a wide range of antibiotics by both *S. aureus* and CoNS represents a significant threat to future treatment efficacy of staphylococcal infections. The issue of drug resistance in this group of pathogens needs to be addressed via appropriate use of existing drugs as well as the development of novel agents [8,11]. Multidrug-resistant staphylococci are potential targets for bacteriocin applications, including lysostaphin.

## 3. Lysostaphin General Features

Class III bacteriocins include large peptides (*M*_r_ ≥ 25 kDa) which are generally heat-labile. This class of bacteriocins was further subdivided by Heng and co-workers into two distinct groups: (i) the bacteriolytic enzymes (or bacteriolysins) and (ii) the non-lytic antimicrobial proteins [1]. Staphylococci have been shown to produce bacteriolysins, from which lysostaphin is considered to be the prototype. Lysostaphin, an extracellular enzyme secreted by strains of *S. simulans* biovar *staphylolyticus* [12], was probably the first staphylococcin (bacteriocin produced by staphylococci) discovered. Its bactericidal activity against staphylococci relies on its capability of cleaving the peptidoglycan present in the bacterial cell walls.

**Figure 1:**
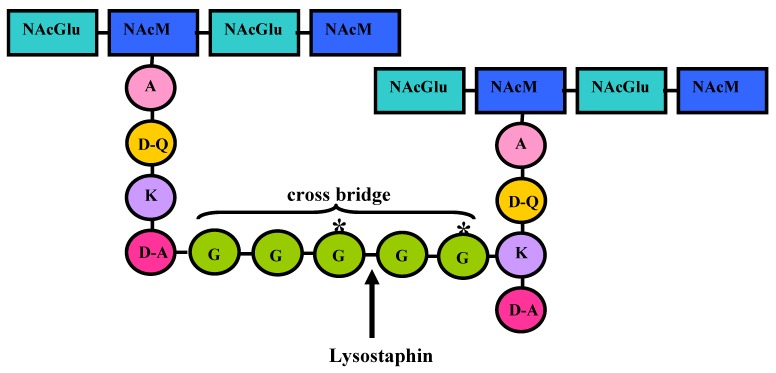
*S. aureus* peptidoglycan structure and site of primary hydrolysis of lysostaphin on the staphylococcal peptidoglycan. NacGlu, *N*-acetylglucosamine; NacM, *N*-acetylmuramic acid; A, L-alanine; D-Q, D-glutamine; K, L-lysine; D-A, D-alanine; G, L-glycine.

The peptidoglycan of Gram-positive microorganisms is an important component of the bacterial cell wall, conferring strength and rigidity to the cell, allowing growth and division, maintaining cell shape, and protecting against osmotic lysis [12]. In *S. aureus*, it consists of a backbone made up of alternating β-1,4 linked *N*-acetylglucosamine and *N*-acetylmuramic acid residues. Muramic acid residues are cross-linked by tetrapeptide chains consisting of D-alanine, D-isoglutamine, L-lysine, and D-alanine. These tetrapeptide chains are cross-linked by pentaglycine bridges between the ε-amino group of the lysine residues of one chain and the D-alanyl carboxyl group of another chain, resulting in a network of two or three dimensions (Figure 1). The products of the genes *fmhB* and *femAB* form this polyglycine bridge. FmhB is involved in the attachment of Gly 1 to the pentaglycine interpeptide. FemA is responsible for the incorporation of Gly 2 and 3, while FemB performs the introduction of Gly 4 and 5 [13,14]. The extreme mechanical strength of *S. aureus* cell walls is probably dependent on the high degree of cross-linking of the pentaglycine bridges between adjacent tetrapeptides. The peptidoglycan is insoluble due to this cross-linking of the polymers and hydrolysis of any single chemical linkage in sufficient number within the cross-linked network can bring about solubilization of the cell wall [15]. However, during physiological growth of the cell wall, the peptidoglycan is hydrolyzed at specific times and sites [16]. This is accomplished by murein hydrolases and lysostaphin is one example of such class of enzymes. In general, murein hydrolases harbor an N-terminal signal peptide followed by a second domain containing the enzymatic activity [17]. In addition, these proteins harbor repeated sequences that flank either the N- or C-terminal side of the enzymatic domain. 

**Figure 2:**
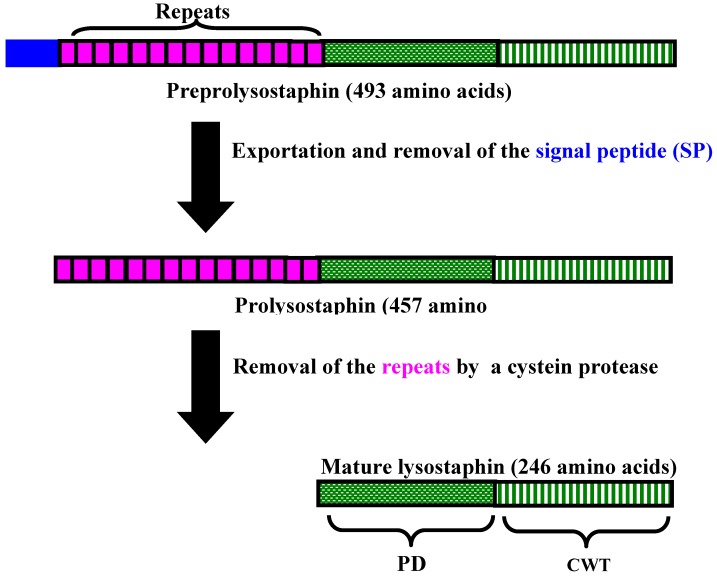
Lysostaphin processing. Lysostaphin is synthesized as a preproenzyme, and after signal peptide removal, soluble prolysostaphin is released into the extracellular medium. Proteolytic cleavage of the propeptide of 211 amino acids, from which 195 residues are organized in 15 tandem repeats of 13-amino acid length, generates the biologically active lysostaphin, which functions as a glycil-glycine endopeptidase against susceptible staphylococcal cells. The lysostaphin molecule consists of two distinct domains: an N-terminal peptidase domain (PD) responsible for its catalytic activity and the C-terminal wall targeting domain (CWT), which directs lysostaphin to its receptor on the staphylococcal surface (reproduced with modifications with kind permission of Bastos *et al*. [6]).

Lysostaphin is a monomeric zinc-containing metallo-enzyme of 246 amino acids. It has a molecular mass of ~27 kDa, a p*I* of 9.5 and a pH optimum of 7.5 [18,19]. Lysostaphin is synthesized as a preproenzyme of 493 amino acids, which is initiated into the secretory pathway by an N-terminal leader peptide of 36 amino acids (Figure 2). The proenzyme is released into the culture medium and contains 15 tandem repeats of 13 amino acid length at the N-terminal end [20]. Prolysostaphin is 4.5-fold less active than mature lysostaphin and the N-terminal repeats are removed in a growth phase-dependent manner by a secreted cystein protease to yield the fully-activated lysostaphin molecule [21]. The lysostaphin molecule consists of two distinct domains: (i) an N-terminal peptidase domain responsible for the catalytic activity of the protein [12] and (ii) a C-terminal targeting domain (CWT) involved in binding to the peptidoglycan substrate [22,23]. The C-terminal 92 amino acid residues of lysostaphin are dispensable for enzymatic activity but necessary and sufficient for directing lysostaphin to the cell wall envelope of *S. aureus*. A study using a reporter system of CWT-GFP (green fluorescent protein) revealed that CWT binds to the pentaglycine bridge bringing lysostaphin into proximity with the cell wall. A *femAB* mutation, reducing both the amount and the length of peptidoglycan cross-linking (resulting in monoglycine cross bridges), showed a dramatic reduction in GFP-CWT binding. Thus the CWT domain of lysostaphin directs the bacteriocin to cross-linked peptidoglycan, which also serves as the substrate for its glycyl-glycine endopeptidase domain [24].

* Replacement of these Gly residues by Ser residues results in resistance to lysostaphin. (reproduced with kind permission of Bastos et al. [6], with modifications)

A preprolysostaphin variant, smaller than that described above, has been reported in the literature, although the number of amino acid residues in the mature lysostaphin remained the same [25].

Lysostaphin production occurs in stationary-phase cultures of *S. simulans* grown under certain conditions and appears to be coordinated with production of other extracellular enzymes [26]. Lysostaphin producers do not have specific immunity genes, but rely on a resistance mechanism (see below) to survive during lysostaphin biosynthesis. 

The lysostaphin endopeptidase gene (*lss*) and the gene involved in lysostaphin resistance (*lif*) reside on plasmid pACK1 [21,27,28]. *lss* and *lif* are flanked by insertion sequences, suggesting that *S. simulans* biovar *staphylolyticus* may have received these genes by horizontal gene transfer [21].

The *lss* gene has been cloned and expressed heterologously in *Escherichia coli* [25], in a simian kidney cell line [29], and in *Lactococcus lactis* [30], a microorganism with a high biotechnological potential. The *lss* gene heterologous expression has advantages when compared to the homologous one: the amount of lysostaphin produced could be improved and the precise moment of bacteriocin production could also be tailored by using inducible-production systems. Using the NICE (NIsin-Controlled gene Expression) system, based on the regulation mechanism of the nisin operon [2], Mierau and co-workers produced lysostaphin in industrial scale obtaining a yield of 300 mg/L [30,31].

A homolog of lysostaphin, ALE-1 *from Staphylococcus capitis*, has also been described and characterized. Its molecular structure and targeting mechanism, as well as the producer resistance, appear to be similar to those described for lysostaphin [32,33].

## 4. Lysostaphin mode of action

Lysostaphin passes freely through the capsule layer of encapsulated staphylococci [34] and is a cell-wall lytic enzyme. The cell-wall degrading activity of lysostaphin is primarily due to a glycyl-glycine endopeptidase activity (Figure 1), which lyses many staphylococcal strains [35]. Lysostaphin has been described as primarily active against CoPS [36], retaining some residual activity against CoNS, although CoNS seem to require increased concentration of the enzyme and larger incubation times to be killed [37]. Studies performed in our laboratory, however, have shown that lysostaphin exhibits an *in vitro* inhibitory activity against strains of many staphylococcal species, including CoNS (Table 2). 

**Table 2:** Antimicrobial spectrum of lysostaphin against *Staphylococcus* spp. strains.

Indicator Strains	Origin	Inhibition Zones
*S. aureus*		
MB269, MB274, MB276, MB277, MB288, MB289, MB295, MB302, MB303		
	Bovine mastitis; Brazil	+++
2, 3, 7, 9, 10	Bovine mastitis; Argentina	+++
3H1, 13H1	Salad	+++
6H4, 13S2	Salad	++
LI1, A70, A53	Pasteurized milk	+++
LF2, LIN4	Sausage	+++
Q2, QRFH1	Cheese	+++
S. carnosus* CN83	Meat fermentation product	++
S. epidermidis*		
CN69	Blood	++ (t)
CN72	Blood	+++
S. haemolyticus*		
CN61	Blood	++
CN68	Blood	-
S. saprophyticus*		
CN86	Urine	++
CN88	Fistula	++
*S. hominis* CN70*	Blood	++
*S. simulans* CN87*	Blood	+++
*S. xylosus* CN93*	Skin	+++
*S. hyicus* ATCC 11249*	-	+++
*S. intermedius* ATCC 29663	-	++

The spectrum of activity was tested by the deferred-antagonism test in BHI agar plates using a strain of *S. simulans* which produces lysostaphin. -, no activity; ++, inhibition zones between 10 and 19 mm; +++, inhibition zones larger than 19 mm; (t), turbid zone of inhibition. The inhibition zones disappear upon treatment with proteolytic enzymes. All strains marked with an asterisk are coagulase-negative staphylococci.

The target of the lysostaphin is the pentaglycine cross-bridge of the peptidoglycan [38], which in *S. aureus* and other staphylococcal species is composed of five glycine (Gly) residues [39,40]. Lysostaphin seems to cleave specifically between the third and the fourth Gly residues of the pentaglycine cross-bridge (Figure 1) [41]. The peptidoglycan of staphylococcal species relatively resistant to lysostaphin contains higher amount of serine (Ser) than Gly [15].

In a study published in 2008, Francius and co-workers [42] used atomic force microscopy (AFM) to track the structural and physical dynamics of single *S. aureus* cells exposed to lysostaphin. They observed that, after being incubated with 16 mg/mL of lysostaphin for a period of 260 min, the cells suffered a length increase of 100 nm. Such cell swelling was taken as an evidence for the formation of lysostaphin-induced osmotically fragile cells, resulting from peptidoglycan hydrolysis. Progressive alteration of the cell surface structure following lysostaphin addition was also observed. After 80 min, nanoscale perforations were seen, which were about 50 to 100 nm in diameter and 25 to 75 nm in depth. With time (e.g., after 260 min), these holes enlarged until they merged together to form larger perforations. These structures were related to reminiscent of the small depressions seen at the onset of normal cell division and could reflect the so-called murosomes, *i.e.*, regions of the cell wall having high autolytic activity. Besides localized surface modifications, it was also found that lysostaphin increased the cell surface roughness. Progressive disintegration of the cell wall and separation from the plasma membrane after exposure to lysostaphin were also observed. These time-dependent cell wall modifications may result from peptidoglycan digestion, eventually leading to the formation of osmotically fragile spheroplasts, favoring cell lysis. Therefore, by hydrolyzing the cell wall, lysostaphin kills the sensitive cells.

**Figure 3:**
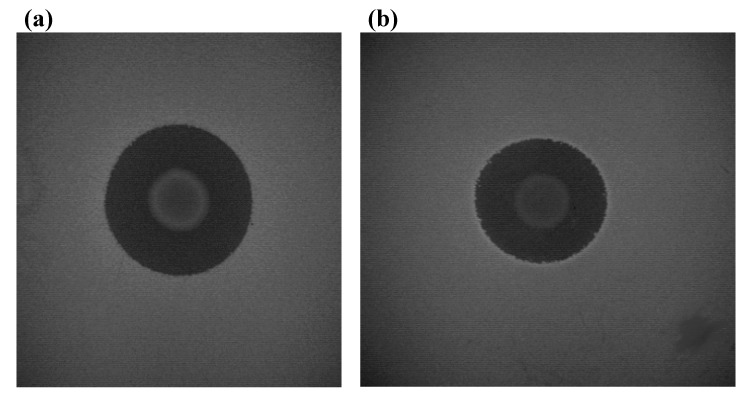
Inhibition of staphylococcal strains by lysostaphin tested by the deferred-antagonism test in BHI medium. (a) *S. aureus* QRFH1, isolated from cheese (inhibition zone of 22 mm). (b) *S. xylosus* CN93, isolated from human skin (inhibition zone of 19.5 mm).

Two basic procedures applied to bacteriocins in general may be used to investigate the lysostaphin spectrum of activity: the simultaneous-antagonism test and the deferred-antagonism test [1]. Briefly, simultaneous-antagonism testing involves stab inoculation of the lysostaphin-producing strain into a freshly seed lawn of a target microorganism (~10^6^ CFU). Following incubation for 18 h, the plate is examined for zones of growth inhibition surrounding the bacteriocin-producing culture. For deferred-antagonism testing, the producing strain is grown as a spot on the surface of an agar plate and then, after sterilization of the agar surface with either chloroform vapor or UV light, the plate is sprayed with the target strain (~10^6^ CFU). Following incubation for 24 h, the extent of inhibition of the target strain by the producing strain can be assessed and the inhibition zones measured (Figure 3).

When a partially or totally purified lysostaphin preparation is available, the critical dilution assay may also be employed. In this method, a 100-μL volume of lysostaphin fractions at two-fold dilutions, and a 100-μL volume of the target strain (~5×10^5^ CFU) are added to each well of a microtiter plate. Dilutions of lysostaphin should be prepared in medium containing 0.1% bovine serum albumin to prevent adsorption of lysostaphin to polystyrene microtiter wells [43]. After incubation at 37 ^o^C for 24 h, the growth of the target strain is measured spectrophotometrically at 600 nm. One bacteriocin arbitrary unit is defined as the amount of bacteriocin that inhibits the bacterial growth by 50% (50% of the turbidity of the control culture without bacteriocin) [44]. Alternatively, a disk-diffusion assay (50 μg lysostaphin/disk) may be used [45] and staphylococcal strains susceptible to lysostaphin give inhibition zones ≥11 mm.

*S. simulans* biovar *staphylolyticus* peptidoglycan is resistant to the hydrolytic activity of lysostaphin, since the cells produce a resistance factor that causes the incorporation of Ser residues into the third and fifth positions of the cell wall cross bridges (Figure 1) [21,46,47]. This incorporation requires the presence of FemA and/or FemB [47]. If one or more Gly residues of the cross bridge are replaced by Ser residues, the cell wall becomes less susceptible to lysostaphin [21,48]. Lysostaphin is unable to hydrolyze glycylserine and serylglycine peptide bonds [48]. It was also shown that lysostaphin binds much more avidly to *S. aureus*, which possesses a pentaglycine cross-bridge, than to *S. simulans* cells. When added to mixed bacterial populations, purified lysostaphin kills 1,000 *S*. *aureus* cells for every *S. simulans* cell [23].

The *S. simulans* gene *lif* is required for incorporation of Ser residues into the peptidoglycan [21]. *Lif* shows similarity to FemA and FemB proteins [21], which catalyze the non-ribosomal synthesis of the pentaglycine cross bridges of the staphylococcal peptidoglycan [13].

## 5. Lysostaphin Potential Biotechnological Applications

The primary role of bacteriocins is to provide the producing bacteria with an advantage over their competitive organisms occupying the same ecological niche, especially in Gram-positive microorganisms, whose bacteriocins may exhibit a broad spectrum of activity directed primarily against other Gram-positive organisms [1]. However, this antagonistic activity may be exploited for biotechnological applications, representing an alternative to antibiotics in either the prevention or the treatment of bacterial infections. As it will be explained to the reader in the next sections, the bacteriocin lysostaphin possesses many characteristics that make it a promising candidate for biotechnological applications.

### 5.1. Research Applications

A recombinant lysostaphin expressed in *E. coli* is sold commercially by Sigma-Aldrich and is indispensable for staphylococcal genetic studies, being used for DNA isolation [49], formation of protoplasts, and differentiation of staphylococcal strains [50].

### 5.2. Human Medical Use

Due to lysostaphin ability to kill human pathogenic staphylococci, such as *S. aureus* and *S. epidermidis*, various reports from the 1960s and 1970s have recommended its use against staphylococcal infections. However, study of lysostaphin as an antistaphylococcal agent was discontinued due to the lack of homogeneous preparations of lysostaphin and the availability of other effective treatments. With the rapidly decreasing effectiveness of current antibiotics for treatment of staphylococcal infections and the availability of recombinant lysostaphin (r-lysostaphin), studies investigating lysostaphin as a therapeutic agent for staphylococcal infections have emerged. Lysostaphin has many attractive features for use as an antimicrobial agent: (i) it has activity against non-dividing as well as dividing cells, (ii) it is digested by intestinal proteinases, having no influence on the gut microbiota, (iii) it has no toxicity, (iv) it is relatively stable when conjugated with polyethylene glycol (PEG), and (v) it maintains its activity in human serum [29,51,52]. Moreover, studies demonstrate that lysostaphin retains its bacteriolytic activity *in vivo*, without any undesirable immune reaction, despite the presence of high-neutralizing antibody titer [53,54].

#### 5.2.1. *In vitro* studies

In 1965, Harrison and Cropp reported the efficacy of lysostaphin over 50 *S. aureus* clinical isolates, most of them penicillinase producers [55]. Lysostaphin was compared to the antibiotics penicillin G, ampicillin, methicillin, ristocetin, vancomycin, and erythromycin. The results have shown that the minimal inhibitory concentration (MIC) values for lysostaphin ranged from <0.047 to 12.5 µg/mL, with 96% of the penicillinase-positive strains being inhibited by 1.56 µg/mL of lysostaphin, whereas 3.12 µg/mL of vancomycin and methicillin were required to attain the same degree of inhibition. Similar findings using other clinical isolates of *S. aureus*, presenting the most diverse drug-resistance patterns, were also reported by other authors that together have tested ~1,000 strains, detecting no resistance to lysostaphin [36,56,57,58]. 

The use of combinations of antimicrobials is common in the clinical setting since it expands the spectrum of organisms that can be targeted, prevents the emergence of resistant organisms, decreases toxicity by allowing lower doses of both agents, and can result in synergistic inhibition. Synergy has been observed *in vitro* between lysostaphin and membrane-active agents, such as polymyxins B [59] and ranalexin [60], a cationic peptide produced the *Rana* frog species, against MRSA.

The increasing use of indwelling intravascular catheters for diagnostics of diseases and for therapeutic procedures has led to an increase in the number of medical device-related infections [9]. Sepsis associated with internal luminal colonization of central venous catheters has been described in many clinical settings and is a frequent occurrence in hospitals today [11,61]. *S. epidermidis* is by far the most frequently isolated species of CoNS from these infections, accounting for 74% to 92% of hospital-acquired CoNS bacteremia [11,62,63]. Therefore, lysostaphin was tested as a preventive agent in surface colonization by either *S. aureus* or *S. epidermidis*. Wu and co-workers [64] reported that lysostaphin not only killed *S. aureus* in biofilms but also disrupted the extracellular matrix of *S. aureus* biofilms *in vitro*, on plastic and glass surfaces, at concentrations as low as 1 µg/mL. Scanning electron microscopy confirmed that lysostaphin eradicated both the sessile cells and the extracellular matrix of the biofilm. For *S. epidermidis*, higher concentrations of lysostaphin were needed to achieve the same effect. In another study, Shah and co-workers [65] tested lysostaphin over different surfaces (polystyrene plates and fluoroethylene-propylene polymer catheters). These surfaces were challenged with an inoculum of about 10^4^ colony-forming units (CFU) of *S. aureus*. On average, 610 CFU were recovered from the polystyrene control wells, whereas only 3 CFU remained in the lysostaphin-coated wells, a 99.5% reduction in bacterial counts. The lysostaphin-coated catheters were completely cleared of bacteria as compared to control catheters, from which an average of 493 CFU were recovered. The inhibitory effect of lysostaphin-coated catheters was maintained for at least four days after coating. Both these studies suggest that lysostaphin binds to plastic surfaces, maintaining killing activity against staphylococci, and may protect catheters from staphylococcal colonization at the time of insertion and for several days thereafter. 

#### 5.2.2. *In vivo* studie*s*

Experimental *in vivo* applications of lysostaphin are also well documented in the literature. The first report of efficacy of lysostaphin against *S. aureus in vivo* was published by Schuhardt and Schindler in 1964 [66]. They tested lysostaphin therapy in mice infected with *S. aureus*. Therapy consisted of a single injection of a partially purified preparation of lysostaphin either intraperitoneally or subcutaneously. These experiments indicated that 10 U of lysostaphin (one unit was defined as the amount required to bring about a 50% reduction in the turbidity of a standard suspension of *S. aureus* FDA 209P in 10 min at 37 °C) by either route of injection cured all mice. 

Abscesses are the hallmark of staphylococcal disease and many therapeutic failures appear to result from the inability of available antimicrobials to eliminate staphylococci residing in high titers in abscess lesions. Although antibiotics do penetrate abscess cavities in high concentration, it has been shown that these agents are relatively ineffective against sluggishly multiplying bacteria found in such lesions [67]. Since previous studies had shown that lysostaphin kills CoPS regardless of their metabolic state [51], Dixon and co-workers [67] tested the effectiveness of lysostaphin (5 mg) followed by treatment with methicillin (5 mg) against established renal abscess lesions in mice. A single dose of lysostaphin, followed by four daily doses of methicillin, produced striking reductions (>99.99%) of the staphylococcal populations. Such reductions were significantly higher than that achieved by either lysostaphin or methicillin alone, although lysostaphin alone proved to be more effective than methicillin alone. Similar results were reported by Harrison and Zygmunt [68].

With the development of r-lysostaphin which is more than 90% pure, it was possible to better study the efficacy of lysostaphin in an experimental *S. aureus* endocarditis model. Studies, using a rabbit model of aortic valve endocarditis, caused by clinical isolates of either MRSA [69] or *S. aureus* with reduced sensibility to vancomycin (VISA) [43], attempted to evaluate the effectiveness of various regimens of dosing with intravenous lysostaphin to reduce the infection. Climo and co-workers [69], using MRSA strains, treated the animals for three days with different doses of r-lysostaphin, vancomycin or a combination of r-lysostaphin and vancomycin. The most active regimen, r-lysostaphin given three times daily, produced sterile vegetations in 10 out of 11 treated rabbits, with a mean reduction in vegetation bacterial counts of 8.5 log_10_ CFU/g compared to the counts in the untreated controls. In contrast, vancomycin given twice daily sterilized no vegetations and reduced vegetation bacterial counts by only 4.8 log_10_ CFU/g. r-Lysostaphin given once daily was less effective, reducing mean vegetation bacterial counts by only 3.6 log_10_ CFU/g, but the combination of r-lysostaphin once daily and vancomycin twice daily reduced the mean vegetation bacterial density by 7.5 log_10_ CFU/g, a result that was significantly better than that for either regimen alone. Yet, when evaluation of the immunological effects of r-lysostaphin administration was carried out, no evidence of immunological reactions following up to nine weeks of intravenous administration could be detected. In the study performed by Patron and co-workers [43], using VISA, it was observed that vancomycin was ineffective, with no evidence of sterilization of aortic valve vegetations. However, rates of sterilization of aortic valve vegetations were significantly better for animals treated with either a single dose of r-lysostaphin (43%) or r-lysostaphin given twice daily for three days (83%) than for animals treated with vancomycin. Rabbits given a single dose of r-lysostaphin followed by a three-day drug-free period had mean reductions in aortic valve vegetation bacterial counts of 7.27 and 6.63 log_10 _CFU/g compared with those for untreated control rabbits and the vancomycin-treated group, respectively. Using a similar experimental approach but with methicillin-resistant *S. epidermidis* isolates (generally less susceptible to lysostaphin) involved in central venous catheter infections, Kiri and co-workers [70] showed that the rabbits treated with the combination of nafcillin (200 mg/kg intramuscularly) and r-lysostaphin (1mg/kg intravenously) had a significant reduction in mean log_10_ vegetation counts (5.32 log_10_ CFU/g) compared to rabbits treated with r-lysostaphin or nafcillin alone. Here again, synergy between r-lysostaphin and β-lactams was observed as already described in previous similar experiments performed with MRSA [71].

More recently, Placencia and co-workers [72] performed a study to evaluate the use of lysostaphin to treat neonatal late-onset sepsis caused by *S. aureus*. This microorganism is the second most common pathogen for late-onset sepsis among very low birth weight infants, and nearly 20% die as a direct result of the infection. In developing countries, neonatal *S. aureus* bacteremia is even more prevalent, causing nearly a quarter of all bacteremic episodes. In their study, Placencia’s group compared lysostaphin *versus* vancomycin against the MRSA strain US300, in a neonatal mouse model. Pups were infected subcutaneously and littermates randomized to receive intraperitoneally either saline (group 1; 81 pups), vancomycin 15 mg/kg (group 2; 77 pups), lysostaphin 10 mg/kg (group 3; 79 pups), and lysostaphin 15 mg/kg (group 4; 75 pups), at time points 0.5, 6, 24 and 30 h after the infection. Pups were observed for survival and growth during seven days, and quantitative blood cultures were obtained 24 h after infection. The survival rates were 6.2%, 34%, 41% and 52%, respectively for groups 1–4. Interestingly, lysostaphin (15 mg/kg; group 4) improved survival compared with placebo and vancomycin. There was no significant difference in growth among the groups and all treatment regimens resulted in less bacteremia when compared with placebo. The MIC and minimum bactericidal concentration (MBC) for vancomycin and lysostaphin were 0.71/1.19 μg/mL and <0.008/0.015 μg/mL, respectively. The serum concentrations after 48 h were of 2.34 μg/mL for lysostaphin and 1.72 μg/mL, for vancomycin. Notably, both serum concentrations were greater than the MICs and MCBs and lysostaphin serum concentrations were higher than vancomycin ones, suggesting a higher half-life for this bacteriocin. These pharmacokinetics data is in agreement with the literature and similar results can be also seen in Oluola *et al*. [73] and Kokai-Kun *et al.* [74]. 

Walsh and co-workers [52], using adult mice as model, compared the pharmacokinetics of r-lysostaphin with a PEG-lysostaphin conjugate (PEGylated). It was found that 24 h after a single dose of 40 mg/kg of r-lysostaphin, the serum drug concentration dropped 500-fold, whereas for the PEGylated derivative the drop was only 10-fold. This reflects an increase in r-lysostaphin stability due to PEG conjugation. This improved retention of r-lysostaphin should reduce the dosing quantities and frequency needed to maintain plasma drug concentrations above therapeutically effective drug concentrations. Maintaining these high levels of r-lysostaphin for longer periods of time may also result in more rapid clearance of bacterial infections and decrease the probability that r-lysostaphin resistance will emerge. 

Eradication of staphylococcal biofilms by lysostaphin has recently been shown to occur also *in vivo* [75].

#### 5.2.3. Reduction of nasal carriage of staphylococci

Nasal carriage of *S. aureus* has been repeatedly shown to have a significant epidemiological link with subsequent development of staphylococcal disease [8]. Clearance of *S. aureus* nasal colonization can reduce the subsequent risk of development of infections by MRSA in addition to reducing the community and hospital spread of these microorganisms [8]. Mupirocin ointment (Bactroban Nasal; 2% mupirocin calcium ointment; SmithKline Beecham) is the current standard of care for clearance of *S. aureus* nasal colonization, but resistance to this antibiotic has emerged [8]. Several other interventions for the clearance of *S. aureus* nasal colonization have also been explored, including Neosporin^®^ ointment (a bacitracin, polymyxin, and neomycin formulation; Burroughs Welcome & Co.).

Using the cotton rat model, Kokain-Kun and co-workers [76] investigated the capacity of three antibacterial agents, lysostaphin, mupirocin, and nisin, a lantibiotic with a potent *in vitro* antistaphylococcal activity, to clear *S. aureus* [methicillin resistant or sensitive strains (MSSA)] nasal colonization. A single application of lysostaphin (actual dose, ~150 μg), formulated in a petrolatum-based cream, dramatically reduced *S. aureus* nasal colonization by both MRSA and MSSA (averages between 3,416 and 4,021 CFU/nose) in 100% of animals tested and eradicated *S. aureus* nasal colonization in 93% of animals. Interestingly, when lysostaphin was administered in PBS, only 33% eradication was achieved. Yet, a single dose of lysostaphin (formulated at 0.5% in a petrolatum-based cream) eradicated MRSA and mupirocin-resistant *S. aureus* from the cotton rat nares, some within 4 h of application, while three doses of mupirocin ointment over three days were required for eradication of MRSA in the same model. Nisin (actual dose, ~1.5 mg) failed in reducing staphylococcal nasal carriage in this model.

The potency of the lysostaphin formulation was also persistent in the nares and maintained its antistaphylococcal activity for at least 24 h after instillation, suggesting that lysostaphin cream could also prevent subsequent *S. aureus* nasal colonization. Lysostaphin cream could therefore be instilled in the nares of an uncolonized patient upon admission to a health care setting and protect the patient from nasal colonization for at least 24 h.

Quickel Jr. and co-workers [77] tested the efficacy of lysostaphin to alter natural *S. aureus* carriage in 95 persistent carriers. In this work, the efficiency of lysostaphin intranasal spray was compared to Neosporin^®^. The treatment was carried out for five days with either agent significantly reducing carriage rates. However, this effect persisted through the 5th day only after therapy with lysostaphin but not with Neosporin^®^. Immunological parameters were also evaluated and, except for a single immediate wheal and flair skin test reaction, no other evidence of adverse reactions to topical lysostaphin was detected. As well, no consistent changes in hemagglutination-inhibition titers to lysostaphin were observed after therapy. Taken together, these studies have shown that lysostaphin appears to be more effective than conventional topical antimicrobial therapy in reducing nasal carriage of staphylococci. 

### 5.3. Veterinary Use

Mastitis, an inflammatory reaction of the mammary gland that is usually caused by microbial infection, is recognized as the most costly disease in dairy cattle [78] due to rejected milk, degraded milk quality, early culling of cows, drug costs, veterinary expenses, and increased labor costs for farmers. In the USA, annual losses are estimated at two billion dollars. This is approximately 10% of the total value of farm milk sales, and about two-thirds of these losses are due to reduced milk production in subclinically infected cows [79]. Although several bacterial pathogens can cause mastitis, *S. aureus* is the major etiological agent. Intrammamary infections caused by *S. aureus* are usually chronic and subclinical in nature. Once *S. aureus* is established in the mammary glands of the animal, it is very difficult to eradicate [78,80]. 

Many drugs have been used for mastitis treatment, but several factors, including the ability of *S. aureus* to survive inside neutrophils, to induce formation of microabscesses, and its resistance to the antibiotics used for treatment, result in infections that are difficult to manage therapeutically [80]. Lysostaphin has been suggested to be efficacious in the treatment of bovine mastitis caused by staphylococci [81]. 

The kinetics and therapeutic efficacy of the r-lysostaphin (100 mg, administered over three consecutive p.m. milkings) in *S. aureus* bovine mastitis have been investigated [82]. In this study, 30 Holstein-Friesian dairy cattle in their first lactation were infected with *S. aureus* ATCC 29740 in all quarters. Infections were established and monitored for somatic cell counts and *S. aureus* CFU three weeks prior to subsequent treatment. Infected animals were injected through the teat canal with a single dose of r-lysostaphin (dose response 1 to 500 mg) or after three successive p.m. milkings with 100 mg of r-lysostaphin in 60 mL of sterile phosphate buffered saline. The *in vivo* titration suggested that the minimal effective therapeutic dose was 100 mg of r-lysostaphin: 95% of the quarters cleared the milk of detectable *S. aureus* for a minimum of one milking after the last intramammary infusion. Despite the maintenance of a bactericidal activity in the milk under this therapeutic regimen for up 120 h after initiation of therapy, the majority of the r-lysostaphin-treated quarters relapsed within five to six milkings. The data suggested that r-lysostaphin, although effective at eliminating bacteria present in the milk for as long as an effective therapeutic level could be maintained, was not sufficient to elicit cures. Such observations are most likely related to previous observations with *S. aureus* mastitis that showed that viable intracellular bacteria contribute to the reinfection and r-lysostaphin cannot kill intracellular *S. aureus* [80]. The cure rate of quarters receiving r-lysostaphin (100 mg in sterile phosphate buffered saline, administered after each of three consecutive p.m. milkings) was 20% compared with 29% for sodium cephapirin in saline and 57% for a commercial antibiotic formulation (Cefa-Lak^®^), respectively. However, the authors proposed that an improved formulation of r-lysostaphin could be a safe alternative to antibiotic therapy.

Following this study, Daley and Oldham [83] showed that intramammary infusion of r-lysostaphin failed to elicit significant serum titers in the bovine until 18–21 infusions were administered (total administered dose of 2–3 g of protein). Antibody titers from dairy cattle which did develop an immune response were predominantly of the IgG1 subclass. Dairy cattle with significant antilysostaphin titers showed no deleterious symptoms, such as anaphylaxis, upon subsequent infusion, and these titers did not affect the *in vitro* activity of r-lysostaphin. Intramammary infusion of r-lysostaphin did not elicit any observable effects on the host animal or on the potential efficacy of the recombinant molecule. 

More recently, Wall and co-workers [84] produced transgenic cows expressing a modified lysostaphin gene whose product lacks two glycosylation motifs, which are responsible for rendering lysostaphin inactive when glycosylated by mammalian cells. Cow’s lysostaphin concentrations ranged from 0.9 to 14 μg/mL and remained constant during most of lactation. Interestingly, the transgenic protein was 15% as active as the bacterially derived r-lysostaphin. The milk produced by the highest expressing cow was able to inhibit the growth of several *Staphylococcus* spp., including *S. chromogenes*, *S. hyicus*, *S. epidermidis*, *S. simulans*, and *S. xylosus*. The transgenic cows’ ability to resist infection by *S. aureus* was tested by intramammary infusion of viable bacterial cultures. Of the mammary glands infused, 34 of 48 glands (71%) became infected in nontransgenic animals compared to 3 of 21 glands (14%) in transgenic animals. The cow with the lowest *S. aureus* lytic activity had the highest infection rate (two out of six glands infected); the animal with intermediate activity had a lower infection rate (one out of six glands), and the highest expressing animal appeared to be completely protected. Furthermore, even in infected glands, the severity and duration of infections were less than in nontransgenic animals. Increase in milk somatic cells, elevated body temperatures, and induced acute phase proteins, each indicative of infection, were observed in all of the nontransgenic cows but in none of the transgenic animals. Milk from the highest expressing cow completely blocked growth of *S. aureus* in an *in vitro* lawn assay test. This milk, diluted 8 to 16-fold, exhibited full lytic activity.

## 6. Development of Resistance to Lysostaphin: A Possibility

The clinical application of lysostaphin could be compromised by the development of resistance as already observed for many antibacterial agents. No lysostaphin-resistant mutants were found in studies performed *in vivo* in which treatments with high doses of lysostaphin were employed [43,65]. However, development of resistance to lysostaphin by *S. aureus* has been reported by Strandén and co-workers [85].

The mechanisms involved in resistance to lysostaphin seem to vary, but the most common one involves mutations that affect either the *femA* or *femB* genes. These mutations result in monoglycine or triglycine cross bridges [13,85]. 

Climo and co-workers [71] have demonstrated that resistance to lysostaphin can occur both *in vitro* and in an *in vivo* model of infection with MRSA following prolonged exposure to low concentrations of lysostaphin. Resistance to lysostaphin in such mutants was associated with three characteristics: increased susceptibility to β-lactams, mutations in *femA*, and an altered peptidoglycan structure in which the normal pentaglycine cross bridges were replaced with a single Gly. However, this resistance development was easily circumvented by the co-administration of β-lactam antibiotics. Moreover, the combination of lysostaphin and β-lactams appears to be synergistic. These data suggest that therapeutic trials with lysostaphin should include β-lactam antibiotics to both suppress resistance and promote synergy. 

Other works also describe an intrinsic relationship between *femA*-mediated lysostaphin resistance and β-lactams increased susceptibility [70,85]. It has been demonstrated that the modified penicillin binding protein 2 (PBP 2a), encoded by *mecA* and conferring β−lactam resistance on MRSA strains, is unable to perform its function in the absence of the five-membered interpeptide chains [86]. That is, while the low-affinity PBP 2a is still produced in MRSA strains that become lysostaphin resistant due to mutations to *femA,* PBP 2a can no longer function as a transpeptidase for monoglycine interpeptide chains. The normal PBP 2, however, can use these monoglycine interpeptides, but since β-lactam antibiotics can inhibit PBP 2, the resultant phenotype is methicillin susceptible [87]. 

Morikawa and co-workers [88], using *S. aureus* strain N315 and its derivative mutants affected in *sigB* gene, showed that cell-wall thickness also plays important roles in lysostaphin sensitivity. Cells depleted in *sigB* developed thinner envelopes and demonstrated increased sensitivity to lysostaphin. These cells were approximately 97% more sensitive than the wild-type N315 cells. On the other hand, over expressing *sigB* cells revealed an increased resistance to lysostaphin, being 300% more resistant than normal N315 cells. The increased resistance of these cells was attributed to an expressive increase in cell-wall thickness and evidence supportive of this assumption can be find in the literature. For example, the VISA strain MU50 has also been demonstrated to possess an expanded cell-wall and this characteristic has been intrinsically associated to its vancomycin resistance [89]. In another work, Koehl and co-workers [90], studying the low susceptibility of VISA strains to lysostaphin, found out that besides the enlarged cell-wall phenotype, lysostaphin resistance was also linked to a decreased autolytic activity. They proposed that in intact cells the cell’s autolysins participate in the lysis seen upon lysostaphin treatment and that the defective autolysis of VISA strains is mainly responsible for the poor susceptibility of intact cells to lysostaphin. It appears that changes in the amount or activity of the autolytic enzymes are mainly responsible for the autolysin deficiency, even though some changes in peptidoglycan composition were evident in the walls of the VISA strains. Several autolysin genes are present in *S. aureus. atl* is the gene that encodes the major *S. aureus* autolysin that undergoes post-translation processing to yield a 63-kDa amidase and a 53.5-kDa glucosaminidase. However, in this work, northern blot analysis suggested that the expression of *atl* was significantly reduced in the VISA strains, reinforcing the assumption that these autolysins are responsible for promoting cell lysis upon lysostaphin treatment. 

By transposon mutagenesis, Gründling and co-workers [91] have identified in *S. aureus* another gene*, lyrA* (for lysostaphin resistance protein A), whose inactivation caused a high degree of lysostaphin resistance. *lyrA* encodes a 419- amino acid polypeptide with unknown function. In contrast to the case for *femAB* mutants, transposon insertion in *lyrA* did not cause gross alterations of cell wall cross bridges and did not result in a decrease in β-lactam resistance. Therefore, the resistance phenotype of *lyrA* mutants has proven difficult to explain. Many factors, including minor alterations in peptidoglycan, pentaglycine bridges and other envelope components were proposed as possible explanations for this phenotype.

Despite all of these reports about development of lysostaphin resistance among *S. aureus* strains, an important observation was made recently by Kusuma and co-workers [92], which have demonstrated that the development of lysostaphin resistance, due to mutation in *femA*, by two strains of MRSA also led to a loss of fitness in the mutants. The diminished fitness was reflected by: (i) a reduced logarithmic growth rate, with mutants being outcompeted in co-cultures by their wild-type parental strains; (ii) increased susceptibility to elevated temperatures, and (iii) at least fivefold less virulence of the mutants than their wild-type strains in a mouse kidney infection model, with the mutants being outcompeted in co-infection with their wild-type parental strains. During a 14-day serial passage without selective pressure, the lysostaphin resistant mutants failed to develop compensatory mutations which restored their fitness. 

Taken together, these results suggest that, should lysostaphin resistance due to an alteration in the FemA function emerge in *S. aureus* during therapy with lysostaphin, the resistant variants would be less fit and less virulent and, in addition, infections with these strains would be easily treatable with β-lactam antibiotics.

## 7. Conclusions

A current understanding of lysostaphin, the only class III bacteriocin so far exploited for clinical applications, has been presented. Examples of its *in vitro* and *in vivo* applications as a therapeutic agent against pathogenic staphylococci, including multiresistant strains, have clearly been shown. However, its broad clinical application waits for standardization of drug formulations either alone or in combination with other antibiotics. Moreover, since *S. aureus* is also a very important pathogen transmitted by food, being involved in numerous outbreaks in several countries, and since strains of this microorganism isolated from different foods have been shown by our group to be inhibited by lysostaphin (Table 2), this bacteriocin might be useful also as a biopreservative in food industry.
